# *AURKA* rs8173 G>C Polymorphism Decreases Wilms Tumor Risk in Chinese Children

**DOI:** 10.1155/2019/9074908

**Published:** 2019-09-15

**Authors:** Tongyi Lu, Li Li, Jinhong Zhu, Jiabin Liu, Ao Lin, Wen Fu, Guochang Liu, Huimin Xia, Tiesong Zhang, Jing He

**Affiliations:** ^1^Department of Pediatric Surgery, Guangzhou Institute of Pediatrics, Guangdong Provincial Key Laboratory of Research in Structural Birth Defect Disease, Guangzhou Women and Children's Medical Center, Guangzhou Medical University, Guangzhou 510623, Guangdong, China; ^2^Kunming Key Laboratory of Children Infection and Immunity, Yunnan Key Laboratory of Children's Major Disease Research, Yunnan Institute of Pediatrics Research, Yunnan Medical Center for Pediatric Diseases, Kunming Children's Hospital, Kunming 650228, Yunnan, China; ^3^Department of Clinical Laboratory, Biobank, Harbin Medical University Cancer Hospital, Harbin 150040, Heilongjiang, China

## Abstract

Wilms tumor is the most common type of renal malignancy in children. Previous studies have demonstrated that single nucleotide polymorphisms (SNPs) in the *AURKA* gene could predispose to several human malignancies. We recruited 145 cases and 531 cancer-free controls to investigate whether *AURKA* gene variants modify Wilms tumor susceptibility. Three *AURKA* SNPs (rs1047972 C>T, rs2273535 T>A, and rs8173 G>C) were genotyped by the Taqman methodology. Odds ratios (ORs) and 95% confidence intervals (CIs) were used to assess the strength of association between *AURKA* SNPs and Wilms tumor risk. We found that only the rs8173 G>C polymorphism was significantly associated with Wilms tumor risk (GC vs. GG: adjusted OR (AOR) = 0.50, 95% CI = 0.35–0.73, *P*=0.0002; GC/CC vs. GG: AOR = 0.60, 95% CI = 0.42–0.88, *P*=0.008). Stratification analysis revealed that rs8173 GC/CC genotypes were associated with Wilms tumor risk among children aged >18 months (AOR = 0.56, 95% CI = 0.34–0.93, *P*=0.024), male children (AOR = 0.54, 95% CI = 0.33–0.90, *P*=0.017), and children with clinical stage III + IV diseases (AOR = 0.56, 95% CI = 0.35–0.90, *P*=0.017). Haplotype analysis indicated that the CAG haplotype was significantly associated with increased Wilms tumor risk. In conclusion, our findings indicated that the *AURKA* rs8173 G>C polymorphism was associated with decreased Wilms tumor risk in Chinese children.

## 1. Introduction

Wilms tumor (WT), also known as nephroblastoma, is the most common renal malignancy in children [1, 2]. It accounts for 6% to 7% of malignant tumors in children under the age of 15 years, with an incidence rate of about 7–10 cases per million in Western countries [3]. The incidence rate of WT in Chinese is around 3.3 cases per million [4]. Over the past 20 years, because of the application of multimodality treatment, including surgery, radiotherapy, chemotherapy, and autologous stem cell transplantation, the overall survival rate of 5 years has increased from 30% to 90% [2, 5–7]. Despite the great achievements in the treatment of WT, the prognosis of nearly 25% of patients with high-risk diseases remains unsatisfying [8]. In addition, about 25% of survivors suffer from the high cost of treatment and the physical torment of some chronic conditions [9, 10].

Previous studies have found that genetic factors contribute to the risk of Wilms tumor and identified a number of genes associated with WT, including Wilms' tumor protein 1 (*WT1*), *β-catenin*, *tumor protein 53* (*TP53*), *catenin beta 1* (*CTNNB1*), and *AMER1* [11–13]. Although recent genome-wide studies on WT have revealed some previously unknown gene mutations implicated with WT, current known genetic variants are not adequate to fully elucidate the pathogenesis of WT [14]. Therefore, it is particularly important to identify more causal genetic variants for WT.

*Aurora kinase A* (*AURKA*) is located at chromosome 20q13.2, consisting of 12 exons [15]. It encodes a centrosome-related serine/threonine kinase found to be overexpressed in many human cancers, including primary colorectal carcinoma, esophageal squamous cell carcinoma, neuroblastoma, and breast and ovarian cancers [16–19]. Overexpression of *AURKA* leads to abnormal centrosome amplification, thereby affecting the stability of the genome and inducing tumorigenesis [16, 20]. Furthermore, inhibition of *AURKA* expression could result in abnormal cell mitosis, eventually leading to cell death [21–23]. Therefore, *AURKA* might be an attractive target for anticancer therapies [24].

In addition, previous studies had found that the *AURKA* gene single nucleotide polymorphism (SNP) was closely related to the risk of several human malignancies [25–27]. Early research demonstrated that the AA genotype of *AURKA* rs2273535 T>A could increase the risk of oral cancer [28]. Studies in recent years have also found that *AURKA* rs2273535 T>A polymorphism could enhance the susceptibility of gastric cancer [15]. Furthermore, there were related studies reported that Caucasians carrying *AURKA* rs1047972 T>C had a relatively low risk of developing breast cancer [26]. However, studies regarding their association with WT risk are lacking. Therefore, we performed this case-control study with 145 cases of WT and 531 control subjects to assess the association between *AURKA* gene polymorphisms and WT susceptibility in Chinese children.

## 2. Materials and Methods

### 2.1. Study Subjects

In this study, a total of 145 cases of WT and 531 controls were collected from the Guangzhou Women and Children Medical Center from 2001 to 2016, as we described previously [29]. Controls were selected from those receiving routine physical examination during the same period. The cases included in this study were all sporadic and the control groups were confirmed free of history of malignancy. Both WT patients and controls were frequency-matched based on the age, gender, and ethnicity. The frequency distribution information of selected variables for WT cases and cancer-free controls was summarized in Supplementary [Supplementary-material supplementary-material-1]. We obtained written informed consents from the parents or legal guardians of all subjects. The study protocol was approved by the Institutional Review Board of the Guangzhou Women and Children's Center (Ethical Approval Number: 2018022102).

### 2.2. SNP Selection and Genotyping

The selection criteria for potentially functional polymorphisms have been described in detail previously [30]. Briefly, we choose potential functional polymorphisms in the 5′-flanking region, 5′UTR, 3′UTR, and exons of the *AURKA* gene. Three SNPs in the *AURKA* gene were finally selected (rs1047972 C>T (Assay ID: AN322ZF), rs2273535 T>A (Assay ID: C__25623289_10), and rs8173 G>C (Assay ID: C__8947675_10)). The rs1047972 C>T polymorphism may lead to amino acid alteration from Val to Ile at codon 57; the rs2273535 T>A polymorphism may lead to splicing alteration, whereas the rs8173 G>C polymorphism may lead to miRNA binding alteration. These three SNPs could capture additional fourteen SNPs (Supplementary [Supplementary-material supplementary-material-1]). Moreover, there was no significant linkage disequilibrium between paired polymorphisms in Supplementary [Supplementary-material supplementary-material-1] (rs8173/rs1047972, *R*^2^ = 0.119; rs8173/rs2273535, *R*^2^ = 0.527; rs1047972/rs2273535, *R*^2^ = 0.291) [31]. Human genomic DNA was mainly extracted from the peripheral blood samples using the TIANamp Blood DNA Kit (TianGen Biotech, Beijing, China). The mean yield of DNA was 12.21 *μ*g (median = 6.25 *μ*g; range = 1.08 to 60 *μ*g), with a mean A260/A280 ratio of 1.89 (median = 1.90; range = 1.78 to 2.05). *AURKA* SNPs were genotyped using the ABI-7900 Sequence Detection System (Applied Biosystem, Foster City, CA) and Taqman real-time PCR, as described previously [30]. The information on all the genotyped samples for all three SNPs was summarized in Supplementary [Supplementary-material supplementary-material-1].

### 2.3. Genotype and Gene Expression Correlation Analysis

The FAM210B mRNA expression data were retrieved for transformed fibroblasts cells with different genotypes of *AURKA* SNPs from the GTEx Portal database (https://www.gtexportal.org/home/). These data were used to assess the correlation between rs8173 G>C genotypes and mRNA expression-level alteration [32].

### 2.4. Statistical Analysis

All statistical analysis of data was processed by SAS (version 9.4; SAS Institute, Cary, NC, USA). The distribution of subject characteristics between cases and controls was examined by a bilateral *χ*^2^ test. The goodness-of-chi-squared test was used to validate whether the genotype frequencies in the controls were consistent with the Hardy–Weinberg equilibrium (HWE). Multivariate logistic regression analysis was adopted to calculate the odds ratios (ORs) and 95% confidence intervals (CIs). The adjusted ORs and 95% CIs were calculated using an unconditional logistic regression model with adjustment for age and gender to assess the association between *AURKA* polymorphisms and WT risk. Inferred haplotypes of *AURKA* gene were based on observed genotypes, and adjusted ORs and 95% CIs were obtained by logistic regression models with adjustment for age and gender. *P* < 0.05 was considered to be statistically significant.

## 3. Results

### 3.1. Association between *AURKA* Gene Polymorphisms and Wilms Tumor Susceptibility

In this study, 143 cases and 531 controls were successfully genotyped. All selected SNP genotypes had a frequency distribution consistent with HWE (rs1047972 C>T, *P*=0.598; rs2273535 T>A, *P*=0.701; rs8173 G>C, *P*=0.272). The genotype frequencies of the case group and control group are shown in Table 1. We found rs8173 G>C polymorphism was significantly associated with decreased WT risk (GC vs. GG: adjusted OR (AOR) = 0.50, 95% CI = 0.35–0.73, *P*=0.0002; GC/CC vs. GG: AOR = 0.60, 95% CI = 0.42–0.88, *P*=0.008).

### 3.2. Stratification Analysis

Subsequently, we explored the association between rs8173 G>C polymorphism and WT risk by stratified analysis. The results of the stratified analysis were based on the age, gender, and clinical stage (Table 2). We found that carriers of rs8173 GC/CC genotypes had a decreased WT risk when compared with GG genotype carriers, among children older than 18 months (AOR = 0.56, 95% CI = 0.34–0.93, *P*=0.024), male children (AOR = 0.54, 95% CI = 0.33–0.90, *P*=0.017), and those with the clinical stage III + IV diseases (AOR = 0.56, 95% CI = 0.35–0.90, *P*=0.017).

### 3.3. Haplotype Analysis

Based on a combined analysis of the three SNP polymorphisms in the *AURKA* gene, eight haplotypes were inferred (Table 3). Compared with the reference CAC haplotype, only CAG haplotype was associated with significant increased WT risk (AOR = 1.99, 95% CI = 1.05–3.77, *P*=0.034).

### 3.4. Genotype-Based mRNA Expression Analysis

**Figure 1:**
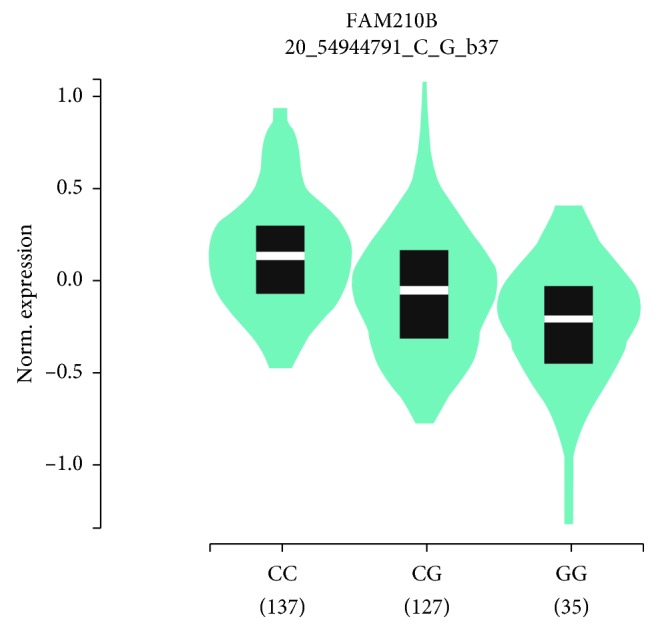
Genotype-based mRNA expression alteration in transformed fibroblasts cells for *AURKA* rs8173 G>C polymorphism based on data from the GTEx portal database (https://www.gtexportal.org/home/).

We found that the rs8173 G>C polymorphism was significantly associated with altered *AURKA* gene expression in transformed fibroblast cells (*P*=4.3 *∗* 10^−12^) using data from GTEx portal (Figure 1).

## 4. Discussion

In this case-control study, we analyzed 143 WT patients and 531 controls to investigate the association between three *AURKA* SNPs (rs1047972, rs2273535, and rs8173) and WT risk of in Chinese children. To the best of our knowledge, this is the first study to investigate the association between *AURKA* gene polymorphisms and WT susceptibility.

Previous studies have found that *AURKA* was overexpressed in several common human malignancies, which in turn promoted cell proliferation and tumor progression and metastasis [33–35]. The abnormal expression of *AURKA* could lead to abnormal chromosome segregation, decreased chromosome stability, and finally increase the susceptibility to malignant transformation [36]. Studies in recent years have shown that *AURKA* SNPs were closely related to cancer risk. An early study by Lee et al. indicated that the *AURKA* gene rs2273535 was associated with oral cancer risk [28]. A research by Guo et al. [37] illustrated that the rs2273535 polymorphism was closely related to the increased risk of breast cancer, especially in Asian populations. Interestingly, studies have also found that the Phe allelic variation in *AURKA* rs2273535 appears to prevent breast cancer in Malaysian Chinese [38]. In addition, Dai et al. [26] reported that the *AURKA* rs1047972 polymorphism was associated with reduced incidence of breast cancer in Caucasians. These findings suggest that the effects of *AURKA* SNPs might be tissue dependent and ethnicity dependent.

We previously did not find a significant association between *AURKA* SNPs and neuroblastoma susceptibility [31]. Interestingly, in this study, our results showed that *AURKA* rs8173 G>C significantly reduced the WT risk, although no significant association was found for *AURKA* rs1047972 C>T and rs2273535 T>A. In addition, stratified analysis revealed that individuals carrying *AURKA* rs8173 GC/CC genotypes had significantly decreased susceptibility to WT in several subgroups, including older than 18 months, male, and clinical stages III + IV.

A previous study showed that the *AURKA* rs2273535 polymorphism had a strong LD with the rs1047972 genotype. Patients with *AURKA* haplotype variants exhibited high kinase activity and tended to develop advanced gastric cancer more readily [39]. In this study, we found that the WT risk in individuals with rs1047972/rs2273535/rs8173 CAG haplotype almost doubled, when compared with individuals with CAC haplotype. However, there were no significant association between the other six haplotypes and the risk of WT.

*AURKA* has been extensively investigated in neuroblastoma. ShRNA-mediated *AURKA* gene silencing assays have demonstrated that reducing *AURKA* expression would inhibit cell proliferation in neuroblastoma [40–42]. It is worth mentioning that studies also have found that overexpression or expansion of *AURKA* is associated with poor prognosis in a variety of cancer patients and inhibition of *AURKA* expression can trigger tumor cell death [21–23]. Previous studies have shown that the LIN28B-RAN-AURKA axis was involved in the development of neuroblastoma, and *AURKA*, as a confluence of LIN28B-RAN signaling, could further promote cell cycle progression by phosphorylating many cell cycle regulators and stabilizing N-myc protein (encoded by *MYCN* gene) [41, 42]. The LIN28B-RAN-AURKA-MYCN signaling cascade in the development of neuroblastoma provides a new insight into the molecular mechanism by which *AURKA* rs8173 reduced the risk of WT. The role of *AURKA* in the development of Wilms tumor is currently lacking. However, based on literature search, we found that *AURKA* is involved in a variety of tumors. Because of the universal contributions of *AURKA* in tumors, we hypothesized that *AURKA* might be also implicated in WT and therefore performed the current study. Based on previous reports, we speculated that the *AURKA* rs8173 might reduce the risk of WT by inhibiting the proliferation and migration of tumor cells through the LIN28B-RAN-AURKA-MYCN signaling pathway.

Some shortcomings should be mentioned in our research. First, the study included 145 patients and 531 controls. Because of the limited sample size, some important results may be accidental. Studies with large samples are indispensible to verify our results. Second, only three *AURKA* SNPs were investigated in the study. Other potentially functional *AURKA* gene polymorphisms should be studied in the future. Third, the genotype distribution in this hospital-based study might not represent genotype distribution across the general population, which may bias the case-control study to some extent. Finally, functional experiments should be conducted to strengthen the findings in the current study.

## 5. Conclusions

Overall, our study confirmed that the *AURKA* rs8173 G>C polymorphism is associated with reduced WT risk in Chinese children. In the future, multicenter collaboration is needed to further expand the sample size from different regions and different races to clarify the impact of *AURKA* SNPs on the risk of WT more accurately.

## Figures and Tables

**Table 1:** Association between *AURKA* gene polymorphisms and Wilms tumor susceptibility.

Genotype	Cases (*N* = 143)	Controls (*N* = 531)	*P* ^a^	Crude OR (95% CI)	*P*	Adjusted OR (95% CI)^b^	*P* ^b^
rs1047972 C>T (HWE = 0.598)							
CC	110 (76.92)	412 (77.59)		1.00		1.00	
CT	30 (20.98)	110 (20.72)		0.89 (0.57–1.39)	0.613	0.89 (0.57–1.39)	0.622
TT	3 (2.10)	9 (1.69)		1.09 (0.29–4.08)	0.898	1.10 (0.29–4.10)	0.891
Additive			0.945	1.05 (0.71–1.55)	0.809	1.04 (0.71–1.54)	0.834
Dominant	33 (23.08)	119 (22.41)	0.866	1.04 (0.67–1.61)	0.865	1.03 (0.66–1.60)	0.889
Recessive	140 (97.90)	522 (98.31)	0.746	1.24 (0.33–4.65)	0.747	1.22 (0.33–4.59)	0.764

rs2273535 T>A (HWE = 0.701)							
TT	66 (46.15)	234 (44.07)		1.00		1.00	
TA	65 (45.45)	234 (44.07)		0.82 (0.57–1.16)	0.263	0.82 (0.57–1.17)	0.265
AA	12 (8.39)	63 (11.86)		0.56 (0.29–1.08)	0.084	0.56 (0.29–1.08)	0.085
Additive			0.502	0.88 (0.67–1.17)	0.377	0.88 (0.67–1.17)	0.374
Dominant	77 (53.85)	297 (55.93)	0.656	0.92 (0.63–1.33)	0.656	0.92 (0.63–1.33)	0.659
Recessive	131 (91.61)	468 (88.14)	0.241	0.68 (0.36–1.30)	0.244	0.68 (0.35–1.29)	0.237

rs8173 G>C (HWE = 0.272)							
GG	71 (49.65)	196 (36.91)		1.00		1.00	
GC	54 (37.76)	263 (49.53)		**0.51 (0.35–0.73)**	**0.0003**	**0.50 (0.35–0.73)**	**0.0002**
CC	18 (12.59)	72 (13.56)		0.62 (0.35–1.08)	0.090	0.61 (0.35–1.08)	0.088
Additive			0.018	**0.74 (0.56–0.98)**	**0.033**	**0.75 (0.56–0.99)**	**0.040**
Dominant	72 (50.35)	335 (63.09)	0.006	**0.59 (0.41–0.86)**	**0.006**	**0.60 (0.42–0.88)**	**0.008**
Recessive	125 (87.41)	459 (86.44)	0.762	0.92 (0.53–1.60)	0.762	0.92 (0.53–1.61)	0.781

OR, odds ratio; CI, confidence interval; HWE, Hardy–Weinberg equilibrium. ^a^*χ*^2^ test for genotype distributions between Wilms tumor patients and controls; ^b^adjusted for age and gender.

**Table 2:** Stratification analysis of *AURKA* rs8173 genotypes with Wilms tumor susceptibility.

Variables	rs8173 (cases/controls)		OR (95% CI)	*P*	AOR (95% CI)^a^	*P* ^a^
	GG	GC/CC				
Age (months)						
≤18	34/97	31/136	0.65 (0.37–1.13)	0.127	0.65 (0.38–1.13)	0.128
>18	37/99	41/199	**0.55 (0.33–0.91)**	**0.021**	**0.56 (0.34–0.93)**	**0.024**
Gender						
Female	31/92	33/141	0.70 (0.40–1.21)	0.199	0.70 (0.40–1.22)	0.203
Male	40/104	39/194	**0.52 (0.32–0.86)**	**0.011**	**0.54 (0.33–0.90)**	**0.017**
Clinical stages						
I + II	25/196	28/335	0.66 (0.37–1.16)	0.144	0.71 (0.40–1.26)	0.245
III + IV	41/196	40/335	**0.57 (0.36–0.91)**	**0.019**	**0.56 (0.35–0.90)**	**0.017**

OR, odds ratio; CI, confidence interval; AOR, adjusted odds ratio. ^a^Adjusted for age and gender, without the corresponding stratification factor.

**Table 3:** Frequency of inferred haplotypes of the *AURKA* gene based on observed genotypes and their association with the risk of Wilms tumor.

Haplotypes^a^	Cases (*n* = 286)	Controls (*n* = 1062)	Crude OR (95% CI)	*P*	AOR (95% CI)^b^	*P* ^b^
CAC	62 (21.68)	279 (26.27)	1.00		1.00	
CAG	17 (5.94)	36 (3.39)	**2.00 (1.06–3.77)**	**0.033**	**1.99 (1.05–3.77)**	**0.034**
CTC	18 (6.29)	86 (8.10)	0.89 (0.50–1.57)	0.676	0.91 (0.51–1.62)	0.748
CTG	153 (53.50)	533 (50.19)	1.21 (0.88–1.68)	0.240	1.21 (0.88–1.67)	0.249
TAC	8 (2.80)	34 (3.20)	1.00 (0.44–2.25)	0.990	0.99 (0.44–2.24)	0.983
TAG	2 (0.70)	11 (1.04)	0.77 (0.17–3.55)	0.736	0.74 (0.16–3.40)	0.694
TTC	2 (0.70)	8 (0.75)	1.06 (0.22–5.09)	0.945	1.09 (0.23–5.28)	0.913
TTG	24 (8.39)	75 (7.06)	1.35 (0.79–2.30)	0.266	1.34 (0.79–2.29)	0.279

OR, odds ratio; CI, confidence interval; AOR, adjusted odds ratio. ^a^The haplotype order was rs1047972, rs2273535, and rs8173; ^b^obtained in logistic regression models with adjustment for age and gender.

## Data Availability

All the data used to support the findings of this study are available from the corresponding author upon request.

## Abbreviations

WT: Wilms tumor
*WT1*: Wilms' tumor protein 1
*TP53*: *Tumor protein 53*
*CTNNB1*: *Catenin beta 1*
*AURKA*: *Aurora kinase A*
SNP: Single nucleotide polymorphism
HWE: Hardy–Weinberg equilibrium
OR: Odds ratio
CI: Confidence interval.

## Supplementary Materials

Supplementary Materials Supplemental Table 1: frequency distribution of selected variables for Wilms tumor cases and cancer-free controls. Supplemental Table 2: polymorphisms captured by the three included *AURKA* polymorphisms as predicted by SNPinfo online software. Supplemental Figure 1: linkage disequilibrium (LD) analysis for the three included polymorphisms in Han Chinese population consisting of CHB (Han Chinese in Beijing, China) and CHS (Southern Han Chinese) subjects. Supplemental Figure 2: diagram showing all the genotyped samples for the three included polymorphisms.
