# Supplementary material for: AURKA rs8173 G>C Polymorphism Decreases Wilms Tumor Risk in Chinese Children
Source: J Oncol. 2019 Sep 15;2019:9074908. doi: 10.1155/2019/9074908 (PMC6766156; doi:10.1155/2019/9074908)
Supplement: Supplementary Materials — Supplemental Table 1: frequency distribution of selected variables for Wilms tumor cases and cancer-free controls. Supplemental Table 2: polymorphisms captured by the three included AURKA polymorphisms as predicted by SNPinfo online software. Supplemental Figure 1: linkage disequilibrium (LD) analysis for the three included polymorphisms in Han Chinese population consisting of CHB (Han Chinese in Beijing, China) and CHS (Southern Han Chinese) subjects. Supplemental Figure 2: diagram showing all the genotyped samples for the three included polymorphisms. [file 9074908.f1.doc]

**Supplementary Information**

| **Supplemental Table 1**. Frequency distribution of selected variables for Wilms tumor cases and cancer-free controls | | | | | |
| --- | --- | --- | --- | --- | --- |
| Variables | Cases (n=145) | | Controls (n=531) | | *P ^a^* |
|  | No. | % | No. | % |  |
| Age range, month | 1-132 | | 0.07-156 | | 0.725 |
| Mean ± SD | 26.17 ± 21.48 | | 29.73 ± 24.86 | |  |
| ≤18 | 66 | 45.52 | 233 | 43.88 |  |
| >18 | 79 | 54.48 | 298 | 56.12 |  |
| Gender |  |  |  |  | 0.956 |
| Female | 64 | 44.14 | 233 | 43.88 |  |
| Male | 81 | 55.86 | 298 | 56.12 |  |
| Clinical stages |  |  |  |  |  |
| I | 4 | 2.76 |  |  |  |
| II | 49 | 33.79 |  |  |  |
| III | 50 | 34.48 |  |  |  |
| IV | 33 | 22.76 |  |  |  |
| NA | 9 | 6.21 |  |  |  |
| SD, standard deviation; NA, not available.  ^a^ Two-sided χ^2^ test for distributions between Wilms tumor cases and cancer-free controls. | | | | | |

| **Supplemental Table 2**. Polymorphisms captured by the three included *AURKA* polymorphisms as predicted by SNPinfo online software | | | | | | | | | | | |
| --- | --- | --- | --- | --- | --- | --- | --- | --- | --- | --- | --- |
| rs | Chr. | Allele | LDsnp | Pop/LD | TFBS | Splicing  (ESE or ESS) | miRNA  (miRanda) | nsSNP | Allele | Asian | CHB |
| **rs1047972** | **20** | **T/C** | **rs1047972** | **1** | **--** | **--** | **--** | **Y** | **C** | **0.861** | **--** |
| rs2180691 | 20 | A/G | rs2273535 | CHB/0.815 | Y | -- | -- | -- | A | 0.661 | 0.667 |
| **rs2273535** | **20** | **A/T** | **rs2273535** | **1** | **--** | **Y** | **--** | **Y** | **A** | **0.797** | **0.367** |
| rs2298016 | 20 | C/G | rs2273535 | CHB/1 | -- | -- | -- | -- | G | 0.367 | 0.367 |
| rs6024840 | 20 | A/G | rs2273535 | CHB/0.845 | -- | -- | -- | -- | A | 0.325 | 0.369 |
| rs6127737 | 20 | A/G | rs2273535 | CHB/0.904 | Y | -- | -- | -- | G | 0.356 | 0.369 |
| rs911160 | 20 | C/G | rs2273535 | CHB/0.904 | -- | -- | -- | -- | C | 0.677 | 0.649 |
| rs1044377 | 20 | G/T | rs8173 | CHB/0.953 | -- | -- | Y | -- | T | 0.622 | 0.600 |
| rs11698420 | 20 | G/T | rs8173 | CHB/0.807 | -- | -- | -- | -- | G | 0.360 | 0.364 |
| rs1926071 | 20 | G/T | rs8173 | CHB/0.811 | -- | -- | -- | -- | G | 0.624 | 0.633 |
| rs1926074 | 20 | A/G | rs8173 | CHB/0.811 | -- | -- | -- | -- | G | 0.633 | 0.622 |
| rs2209593 | 20 | C/T | rs8173 | CHB/0.811 | -- | -- | -- | -- | C | 0.619 | 0.622 |
| rs4811692 | 20 | A/G | rs8173 | CHB/0.808 | -- | -- | -- | -- | G | 0.386 | 0.411 |
| rs6024833 | 20 | A/G | rs8173 | CHB/0.906 | -- | -- | -- | -- | A | 0.378 | 0.378 |
| rs6024836 | 20 | A/G | rs8173 | CHB/0.927 | -- | -- | -- | -- | A | -- | 0.583 |
| rs6127729 | 20 | C/T | rs8173 | CHB/0.804 | -- | -- | -- | -- | C | 0.369 | 0.384 |
| **rs8173** | **20** | **C/G** | **rs8173** | **1** | **--** | **--** | **Y** | **--** | **G** | **0.657** | **0.578** |
| LD, linkage disequilibrium; TFBS, transcription factor binding sites; ESE, exonic splicing enhancer; ESS, exonic splicing silencer; SNP, single nucleotide polymorphism; CHB, Han Chinese in Beijing, China. | | | | | | | | | | | |

**Supplemental Figure 1**


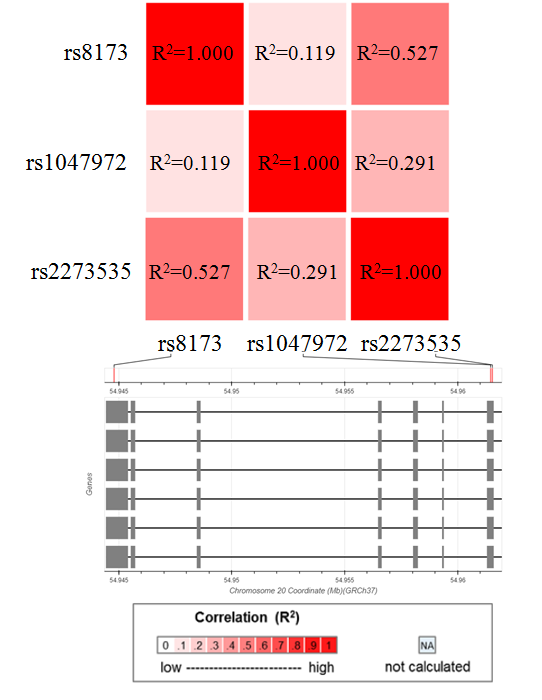


**Supplemental Figure 1**. Linkage disequilibrium (LD) analysis for the three included polymorphisms in Han Chinese population consisted of CHB (Han Chinese in Beijing, China) and CHS (Southern Han Chinese) subjects.

**Supplemental Figure 2**

145 Wilms tumor patients

531 Cancer-free controls

Taqman genotyping for *AURKA* polymorphisms

**rs2273535 T>A**

Cases Controls

(No.) (No.)

TT 66 234

TA 65 234

AA 12 63

2 cases failed

143 Wilms tumor patients

531 Cancer-free controls

**rs1047972 C>T**

Cases Controls

(No.) (No.)

CC 110 412

CT 30 110

TT 3 9

**rs8173 G>C**

Cases Controls

(No.) (No.)

GG 71 196

GC 54 263

CC 18 72

**Supplemental Figure 2**. Diagram showing all the genotyped samples for the three included polymorphisms.
